# Isolated tuberculosis of metacarpal bone in a 3 year-old child

**DOI:** 10.11604/pamj.2017.26.90.11713

**Published:** 2017-02-23

**Authors:** El Mouhtadi Aghoutane, Tarik Salama, Redouane El Fezazi

**Affiliations:** 1 Department of Pediatric Orthopedic Surgery, CHU Mohamed VI, Faculty of Medicine and Pharmacy, University Kadi Ayyad, Marrakech, Maroc

**Keywords:** Tubercular osteomyelitis, metacarpal bone, children

## Abstract

Primary tuberculosis osteomyelitis of metacarpal bone is rare. The majority of cases occur in children and young adults and there is difficulty in diagnosis mainly in young children. We report a new case in children aged of 3 years, presenting a swelling on the dorsal side of her right hand since 8 months. X-ray showed an expansile, cystic and lytic lesion involving the little finger metacarpal. Tuberculosis was confirmed on histological examination. No lesions in lung parenchyma or lymphadenopathy were associated. Patient was successfully managed by anti-tubercular drugs.

## Introduction

Despite the decline in the incidence of tuberculosis during the last decades, the disease remains a significant public health problem in developing countries like Morocco [1]. Metacarpal tuberculosis is a rare presentation of the disease; it represents only 1% of all bone sites [2]. It has an insidious onset and < 50% of patients have active pulmonary disease [2]. We report a new case of metacarpal tuberculosis in a children aged only of 36 months, with literature review.

## Patient and observation

**Figure 1:**
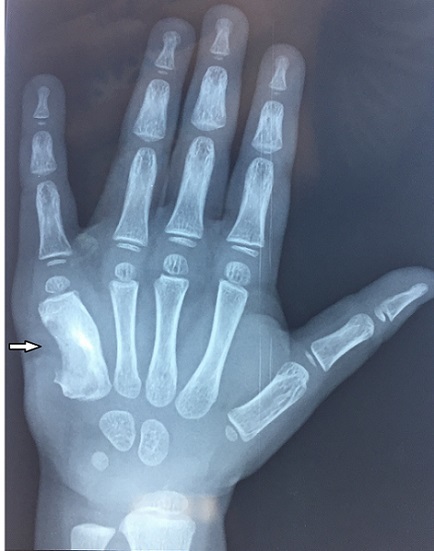
X-ray of right hand showed an expansile, cystic and lytic lesion with cortical erosion involving the little finger metacarpal

**Figure 2:**
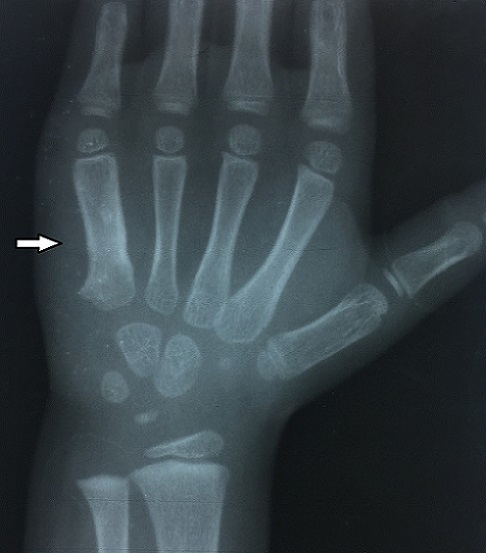
Ray of right hand showed a healing of the lesion after 2 years of evolution

A 36 months old girl of low socioeconomic status presented to our department with eight months history of pain and swelling over right dorsal side of her right hand, with apparition of discharging sinus since 2 months. She was treated by amoxicillin-clavulanic acide for 1 month by another doctor without success. There was no family history of previous exposure to tuberculosis infection and no history of trauma. The boy received BCG vaccination at birth. Physical examination showed normal weight and temperature. Swelling on the ulnar dorsal side of her right hand was found measured 2/2 cm. It was tender to palpation with presence of a narrow draining sinus. Examination of others systems was normal. X-ray showed an expansile, cystic and lytic lesion with cortical erosion involving the little finger metacarpal (Figure 1). The blood investigations revealed anemia (haemoglobin – 10 mg/dl), and acceleration of the erythrocyte sedimentation rate (ESR) of 35 mm/h. white blood cells, and c reactive protein (CRP) were normal. A tuberculin skin test was positive (13/12mm) after 48 hours of test dose. Chest radiograph was normal. The overall appearances were suggestive of chronic osteomyelitis, metacarpal tuberculosis or bony tumor. Histopathology of intra-osseous tissus removed at biopsy confirmed the diagnosis of bone tuberculosis, with typical caseous necrosis surrounded by epithelioid and giant-cell follicles. No organisms were cultured in the purulent materiel, and the Ziehl-Neelson test was negative. Antituberculous treatment was commenced with four drugs (isoniazid, rifampicin, ethambutol and pyrazinamide) for two months, followed by two drugs (isoniazid and rifampicin) for 10 months. At 24 months follow-up, the patient was pain free, the swelling disappeared, and there was no sign of reactivation (Figure 2).

## Discussion

Tuberculosis is an infection disease caused by mycobacterium tuberculosis and is manifested by the formation of tubercles and necrosis in the tissues [2]. Osteoarticular involvement occurs in 1 à 3% patients with extrapulmonary tuberculosis and spine represents 50% of these lesions [3]. Tuberculosis of short bones, like metacarpus, metatarsus and phalanges, is uncommon mainly after the age of 5 years [4]. Isolated metacarpal tuberculosis is much rarer even in endemic areas, and the majority of patients with it are young [3, 4]. Only 1/3^rd^ of patients with tuberculosis of the bone are diagnosed with concomitant active pulmonary disease [3]. Metacarpal tuberculosis in children variedly as painless/painful swelling with/without a discharging sinus [5]. Kotwal et al found pain and swelling to be the most common presenting symptom [6]. While a number of radiographic finding have been identified in association with skeletal tuberculosis, none are pathognomonic [7]. Radiologically the affected metacarpal appears expanded with lytic lesions in the middle (as seen in present case) and subperiostal new bone formation along the involved bone [2, 3]. Differential diagnoses include pyogenic osteomyelitis, enchondroma, sickle cell disease, leutic involvement and boride’s abscess [2, 5, 8]. Inflammatory marker and leukocyte result are often normal, intradermal reaction is usually positive, but when negative, it does not rule out the underlying diagnosis [9, 10]. Diagnosis is confirmed on histology study revealed caseating giant cell granulomas with epithelioid cells [1, 9]. A negative pus culture or inability to see acid fast bacilli under microscope does not exclude tuberculosis [4–6]. The treatment of metacarpal tuberculosis is generally no operatoire [9, 10]. Antitubercular chemotherapy during 12 months (isoniazid, rifampicin, ethambutol and pyrazinamide for two months, then carried on by isoniazid and rifampicin for 10 months) is recommended by the majority of autors [1, 5, 9].

## Conclusion

A very high index of suspicion, early biopsy is required for a timely diagnosis of metacarpal tuberculosis in children. Early commencement Antitubercular chemotherapy was the most important factor for good results. Despite its rarity, the possibility of metacarpal tuberculosis in children should be kept in mind for patients not responding to treatment.
